# A Novel NiFe_2_O_4_/Paper-Based Magnetoelastic Biosensor to Detect Human Serum Albumin

**DOI:** 10.3390/s20185286

**Published:** 2020-09-16

**Authors:** Xing Guo, Rong Liu, Hongmei Li, Jingzhe Wang, Zhongyun Yuan, Wendong Zhang, Shengbo Sang

**Affiliations:** MicroNano System Research Center, Key Lab of Advanced Transducers and Intelligent Control System of the Ministry of Education and College of Information and Computer, Taiyuan University of Technology, Jinzhong 030600, China; guo_xing163@163.com (X.G.); 15235189772@163.com (R.L.); li020622@outlook.com (H.L.); wangjingzhe1234@163.com (J.W.); yuanzhongyun@tyut.edu.cn (Z.Y.); zhangwendong@tyut.edu.cn (W.Z.)

**Keywords:** cellulose paper, NiFe_2_O_4_ nanoparticles, magnetoelastic biosensor, static magnetic permeability

## Abstract

For the first time, a novel NiFe_2_O_4_/paper-based magnetoelastic (ME) biosensor was developed for rapid, sensitive, and portable detection of human serum albumin (HSA). Due to the uniquely magnetoelastic effect of NiFe_2_O_4_ nanoparticles and the excellent mechanical properties of the paper, the paper-based ME biosensor transforms the surface stress signal induced by the specific binding of HSA and antibody modified on the paper into the electromagnetic signal. The accumulated binding complex generates a compressive stress on the biosensor surface, resulting in a decrease in the biosensor’s static magnetic permeability, which correlates to the HSA concentrations. To improve the sensitivity of the biosensor, the concentration of NiFe_2_O_4_ nanofluid and the impregnated numbers of the NiFe_2_O_4_ nanofluid-impregnated papers were optimized. The experimental results demonstrated that the biosensor exhibited a linear response to HSA concentrations ranging from 10 μg∙mL^−1^ to 200 μg∙mL^−1^, with a detection limit of 0.43 μg∙mL^−1^, which is significantly lower than the minimal diagnosis limit of microalbuminuria. The NiFe_2_O_4_/paper-based ME biosensor is easy to fabricate, and allows the rapid, highly-sensitive, and selective detection of HSA, providing a valuable analytical device for early monitoring and clinical diagnosis of microalbuminuria and nephropathy. This study shows the successful integration of the paper-based biosensor and the ME sensing analytical method will be a highly-sensitive, easy-to-use, disposable, and portable alternative for point-of-care monitoring.

## 1. Introduction

Diabetic nephropathy is a chronic complication of diabetes, affecting human endocrine metabolism [1]. There is increasing incidence of diabetic nephropathy in China, and it has become the second cause of end-stage renal disease, second only to various forms of glomerulonephritis. Albuminuria is the first clinical manifestation of diabetic nephropathy, initially intermittent, and then becoming persistent. Elevated albumin levels in urine (20–200 mg∙L^−1^), a condition known as albuminuria, may manifest as renal dysfunction [2]. Detection of the levels of human serum albumin (HSA) or micro human serum albumin (mHSA) in urine can allow earlier diagnosis of albuminuria and can help control the condition [3,4]. HSA is an important human plasma biomacromolecule, accounting for about 60% of total plasma protein, with a molecular weight of 67 kDa [5,6]. Therefore, it is necessary to accurately detect and quantitatively analyze HSA in clinical applications and biochemistry experiments.

In recent years, several methods have been developed for the detection of HSA, including biuret colorimetry [7] and spectral correlation interferometry [8]. Biuret colorimetry offers good stability and repeatability, but this detection method requires measurement of a large number of samples and only low sensitivity [9]. Spectral correlation interferometry has a narrow detection range and poor linearity. Thus, there is a significant need to develop a novel, portable, low-cost HSA detection method with high sensitivity. 

At present, cellulose paper has been developed as a promising platform for biochemical sensors and devices due to its thin, lightweight, and flexible properties [10], which can be integrated in a manner that is inexpensive, portable, disposable, and easy to operate compared to traditional sensors and devices [11,12].

Cellulose paper-based biosensors have several advantages, including having a flat substrate and small pores, and being low-cost, easy to use, and portable, with favorable mechanical flexibility, biocompatibility, and non-toxicity [13,14,15]. Moreover, cellulose paper can be easily functionalized, allowing immobilization with antibodies, or enzymes, or combination with magnetic nanoparticles and noble metal nanoparticles to enhance biosensor performance [16]. In recent years, paper-based sensors have been developed as paper-based electrochemical sensors [17], colorimetric sensors [18], microbial fuel cells [19], and flexible sensors [20]. They are tested by optical and electrochemical methods, but the above methods have some defects, such as the error of test results caused by the illumination difference, and the complicated manufacturing process. At present, the several sensors using magnetic signals have been reported [21,22]. However, to date, most of the research uses the magnetism of magnetic nanoparticles to prepare sensors and detect biological samples by testing optical or electrical signals, motivating a detailed investigation of sensors using magnetic signal.

Magnetoelastic (ME) biosensors are widely used in various fields because of their advantages of high sensitivity and wireless passive monitoring. Among these, the wireless ME sensors whose detection principle is magnetoelastic resonance stand out. They vibrate mechanically in response to a time-varying magnetic excitation field at a particular resonance frequency. Magnetic field telemetry enables contactless, remote-query operation that has enabled many practical uses of this sensor platform. Its capability for detection of gas and humidity concentration, pH measurement, *Escherichia coli*, glucose, avidin, and lipoprotein, among others, has been demonstrated [23]. It has been shown that the control of the geometry of amorphous ribbons is important in order to improve sensibility of the sensor [24], ME microwires [25] are also very promising due to their small size. A portable device for rapid detection of human serum albumin has been developed [26], but its ME biosensor is still based on the Metglas alloy 2826 MB, which has some limitations as the substrate material for biosensors, since it is non-renewable, difficult to biodegrade, high-cost, and has poor mechanical properties.

In this study, cellulose paper is first used as the substrate instead of metal materials to prepare paper-based ME biosensors. Cellulose paper is expected to offer many advantages in the construction of ME biosensors due to its unique properties. First, it can obtain more excellent mechanical properties and reduce the costs of equipment and material; second, it can improve measurement accuracy and realize wireless measurement. Moreover, the study breaks through the limitations previously tested only by the resonant frequency signal [26], and instead testing through magnetic signals.

NiFe_2_O_4_ is a soft magnetic material, a member of the cubic spinel ferrite materials with distinctive chemical properties [27,28], which have facilitated its widespread application in many fields during the past decades. Most importantly, NiFe_2_O_4_ nanoparticles are ME materials with a magnetoelastic effect, which can be used to prepare composite materials for the fabrication of ME biosensors with good electromagnetic energy/mechanical energy conversion properties due to their high energy conversion efficiency. The magnetoelastic effect refers to a phenomenon in which magnetic materials change their magnetic properties under the action of mechanical stress. Therefore, this is also described as an inverse magnetostrictive effect [29]. When a tensile stress acts on the ME material, the magnetization direction is converted into the tensile stress direction, and the magnetization in the tensile stress direction is strengthened, increasing the static magnetic permeability (*μ*) in the tensile stress direction. Compressive stress causes the magnetization direction to turn perpendicular to the stress direction, weakening the magnetization in the compressive stress direction, and reducing the *μ* in the compressive stress direction [30]. The value of *μ* is defined as:

(1) $$\mu =\frac{B}{H}$$

where *H* is the magnetic field strength and *B* is the magnetic induction strength.

Here, a NiFe_2_O_4_/paper-based ME biosensor, which composed of cellulose paper infiltrated with NiFe_2_O_4_ as the transducer platform, and the anti-HSA antibody as the biorecognition molecule, was first developed for HSA detection. For the first time, NiFe_2_O_4_ was doped into the paper, thus changing its properties, so that ordinary paper had magnetoelastic properties. The successful immobilization of anti-HSA antibody provided sites for the highly efficient binding of HSA, which caused the stress variation of the paper-based ME biosensor, inducing the change in the *μ*. The performance of the paper-based ME biosensors was then assessed for different concentrations of HSA. Accumulation of the biomacromolecule HSA on the surface caused a decrease of *μ*. The experimental results showed excellent performance of the paper-based ME biosensor for HSA detection, with advantages of simple preparation method, low cost, high sensitivity, strong specificity, and a large linear range.

## 2. Materials and Methods

### 2.1. Chemicals and Materials

Whatman chromatography paper No.1 was purchased from Zhengcheng Scientific Experimental Equipment Mall. NiFe_2_O_4_ nanoparticles (Chemical Abstracts Service (CAS: 12168-54-6), human serum albumin (HSA, CAS: 70024-90-7) and anti-HSA antibody were purchased from Shanghai Yugong Biotechnology Co., Ltd. Cysteamine (CYS, CAS: 60-23-1), 1-ethyl-3-(3-dimethylaminopropyl) carbodiimide hydrochloride (EDC, CAS: 25952-53-8), N-hydroxysulfosuccinimide (NHS, CAS: 6066-82-6), phosphate buffered saline (PBS, 0.01 M, pH = 7.4) and bovine serum albumin (BSA, 0.1 %, CAS: 9048-46-8) were purchased from Sigma-Aldrich Corporation (Saint Louis, MO, USA). Carcinoembryonic antigen (CEA) was purchased from Beijing Key-Bio Biotech Co., Ltd. Hemoglobin (HGB), uric acid (UA, CAS: 69-93-2), and creatinine (CRE, CAS: 60-27-5) were purchased from Shanghai Fanke Biological Technology Co., Ltd. The water used in the experiments was obtained from an ultra-pure water manufacturing system (URT-11-10T).

### 2.2. Preparation of the Paper

**Figure 1:**
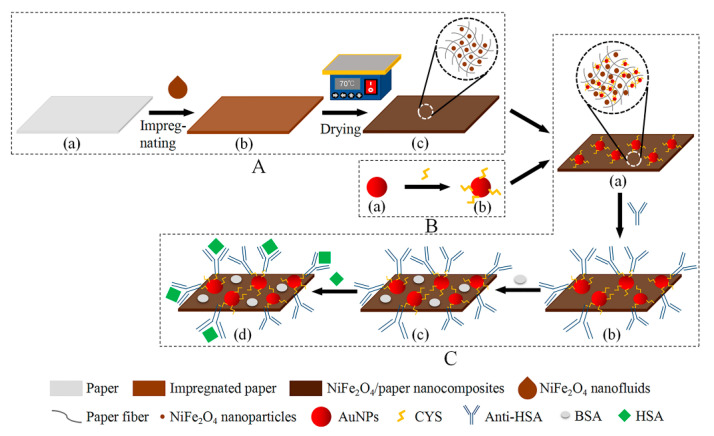
Schematic diagram of preparation and detection procedure of NiFe_2_O_4_/paper-based ME biosensor. (**A**) The fabrication process of the NiFe_2_O_4_/paper nanocomposite, (**a**) paper, (**b**) the NiFe_2_O_4_ nanofluid-impregnated paper, (**c**) the dehydrated NiFe_2_O_4_/paper; (**B**) The fabrication process of the CYS-AuNPs, (**a**) AuNPs, (**b**) CYS-AuNPs; (**C**) The fabrication process of the NiFe_2_O_4_/paper-based ME biosensor, (**a**) CYS-AuNPs/NiFe_2_O_4_/paper-based sensors, (**b**) anti-HSA/ CYS-AuNPs/NiFe_2_O_4_/paper-based sensors, (**c**) BSA/anti-HSA/CYS-AuNPs/NiFe_2_O_4_/paper-based biosensors, (**d**) HSA/BSA/ anti-HSA/CYS-AuNPs/NiFe_2_O_4_/paper-based biosensors.

Different filter papers have been used in the development of paper-based sensors due to their wicking ability [31,32]. Among them, Whatman chromatography paper has been the most widely used. In this study, Whatman chromatography paper No.1 with a thickness of 180 µm and grammage of 88 g/m^2^ was selected as the basis for construction of the NiFe_2_O_4_/paper nanocomposite. Paper of different sizes (16 mm × 1 mm, 4 mm × 4 mm) was prepared for compounding with NiFe_2_O_4_ to obtain NiFe_2_O_4_/paper nanocomposite, as shown in Figure 1A(a). The paper was characterized by SEM, EDS, and FTIR spectra.

### 2.3. Fabrication of the NiFe_2_O_4_/Paper Nanocomposite

Before fabricating the NiFe_2_O_4_/paper nanocomposite, the NiFe_2_O_4_ nanofluid was prepared by dispersing NiFe_2_O_4_ nanoparticles into deionized water. First, NiFe_2_O_4_ nanoparticles were weighed and added into a suitable amount of deionized water. Then, an ultrasonic pulverizer was used for 20 min to fully disperse the NiFe_2_O_4_ nanoparticles into the deionized water to obtain a uniform NiFe_2_O_4_ nanofluid. The NiFe_2_O_4_ nanoparticles were characterized by TEM.

The fabrication process of NiFe_2_O_4_/paper nanocomposite was as follows: (1) Prepared paper samples of different sizes were immersed in the NiFe_2_O_4_ nanofluid for one hour until fully saturated, as shown in Figure 1A(b). (2) The impregnated paper was dehydrated at 70 °C for 10 min on an electric heating plate to achieve a drying effect, as shown in Figure 1A(c). (3) The dried paper sheet was stored in an environment with constant temperature and humidity. The resulting NiFe_2_O_4_/paper nanocomposite samples were characterized by SEM, EDS, and FTIR spectra.

### 2.4. Fabrication of the Paper-Based ME Sensor

First, gold nanoparticles (AuNPs) were prepared. To do this, 250 nL of 0.1 M HAuCl_4_ solution was added to 100 mL of deionized water and heated to boiling. Then, 600 nL of 0.25 M Na_3_C_6_H_5_O_7_ solution was rapidly added to the boiling solution; the color of the solution changed from pale yellow to deep red in a short time, followed by continued heating for 30 min to ensure complete reduction. The obtained solution was centrifuged at 12,000 rpm for 15 min and then washed three times. Finally, the AuNPs were re-dispersed into deionized water and stored at 4 °C (Figure 1B(a)). 

Second, the prepared AuNPs were functionalized. A 4 mM cysteamine solution was mixed with the solution of AuNPs at a volume ratio of 1:9, and then incubated at room temperature for 12 h to obtain thiolated AuNPs (CYS-AuNPs), as shown in Figure 1B(b). The AuNPs and CYS-AuNPs were characterized by TEM and Dynamic Light Scattering (DLS).

Third, the paper-based ME sensors were prepared. Samples of 4 mm × 4 mm NiFe_2_O_4_/paper nanocomposites were horizontally immersed in the solution of CYS-AuNPs for 2 h, and the CYS-AuNPs were fixed on the cellulose fiber and cellulose fiber network of the paper by physical deposition. Finally, the NiFe_2_O_4_/paper nanocomposites with immobilized CYS-AuNPs were dried in a stream of nitrogen to obtain the paper-based ME sensors (Figure 1C(a)).

### 2.5. Antibody Immobilization

The anti-HSA was diluted into PBS buffer (0.01 M, pH = 7.4) to prepare for 50 μg∙ml^−1^ antibody solution [26,33,34]. The anti-HSA antibody was activated through the 4 mg∙mL^−1^—4 mg∙mL^−1^ EDC/NHS mixed solution at room temperature for 30 min. The carboxyl group of the antibody was activated into NHS ester, which make the bonding with the amino group on the CYS-AuNPs be more efficiently [35].

The paper-based ME sensors were immersed into the activated antibody solution at room temperature for 1 h, so that the antibodies were uniformly immobilized on the CYS-AuNPs (Figure 1C(b)). Then, the antibody-modified paper-based ME sensors were removed from the solution and rinsed with PBS to remove physically adsorbed antibodies. Next, the paper-based ME sensors were treated with 0.1 % BSA for 30 min to block the uncombined and non-specific sites (Figure 1C(c)). Then, the paper-based ME sensors were rinsed with PBS and dried with nitrogen, to obtain the paper-based ME biosensor ready for HSA detection.

### 2.6. Signal Measurement

The magnetic field strength of the NiFe_2_O_4_/paper nanocomposites was measured using a Gauss meter. The detection design is shown in Figure 4a. The NiFe_2_O_4_/paper is attached to the tweezer, with one end clamped by adhesive tape. With a tunable external magnetic field produced by adjusting the distance between the NiFe_2_O_4_/paper and a magnet, the tip deflection and bending angle at the free end can be measured. Loading and unloading (hysteresis test) can be achieved by the placement and removal of magnets. 

The hysteresis loop and saturation magnetic induction strength (*B_s_*) of NiFe_2_O_4_ nanoparticles, NiFe_2_O_4_/paper nanocomposites, and paper-based ME biosensors were investigated at room temperature using a vibrating-sample magnetometer (SQUID-VSM, Quantum Design, American) with an applied field of 1.1 T. The VSM test condition requires in-plane dimensions that do not exceed 4 mm × 4 mm, so samples with in-plane dimensions of 4 mm × 4 mm were used to detect the hysteresis loop. During the test of the H we found that long strip samples could more easily detect deflection than squares, so we tested 16 mm × 1 mm samples of the same area to optimize the concentration of NiFe_2_O_4_ nanofluid. In addition, different concentrations of HSA solution were prepared by serial dilution with PBS. The paper-based ME biosensor was immersed in different HSA solutions at room temperature for 1 h, then removed and dried in a stream of nitrogen. Then, the hysteresis loop of the paper-based ME biosensors after detecting different concentrations of HSA (Figure 1C(d)) was investigated. The μ of the materials at maximum magnetic field strength (H_m_) was calculated by Equation (1).

## 3. Results and Discussion

### 3.1. Characterization of NiFe_2_O_4_ Nanoparticles and AuNPs

**Figure 2:**
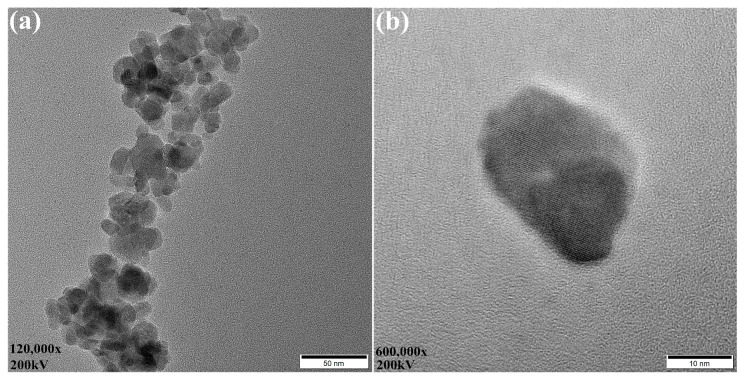
TEM images of NiFe_2_O_4_ nanoparticles. The scale in (**a**) is 50 nm. The scale in (**b**) is 10 nm.

The NiFe_2_O_4_ nanoparticles were characterized using a high-resolution transmission electron microscope (JEM2010-TEM, JEOL, Tokyo, Japan). Figure 2 shows the TEM images of NiFe_2_O_4_ nanoparticles at different multiples. Since NiFe_2_O_4_ nanoparticles have ferrimagnetic properties, the nanoparticles have a large surface energy and are prone to aggregation. As can be seen from Figure 2a, a large number of NiFe_2_O_4_ nanoparticles aggregated into a mist-like cluster, with many spherical particles and a few polygonal particles. The nanoparticles’ size ranged from 20 to 30 nm as shown in Figure 2b.

**Figure 3:**
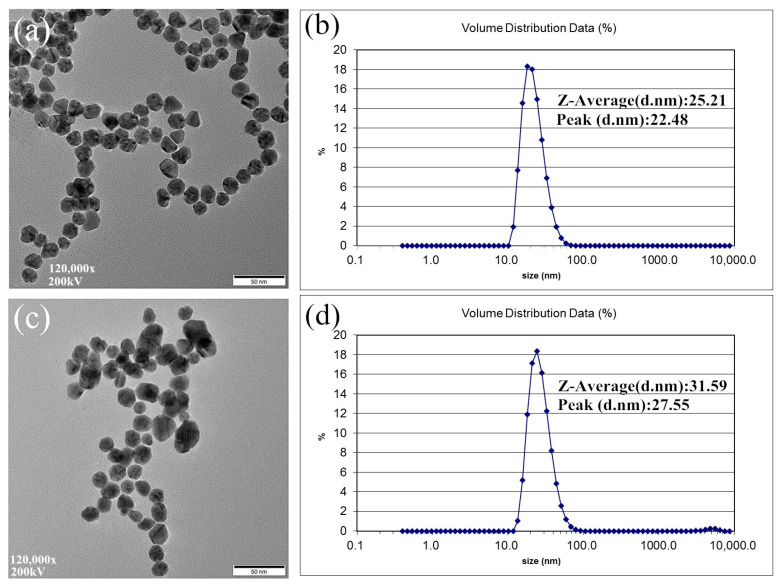
(**a**) TEM image of AuNPs; (**b**) DLS analysis of AuNPs; (**c**) TEM image of CYS-AuNPs; (**d**) DLS analysis of CYS-AuNPs.

Figure 3a,c show the TEM images of AuNPs and CYS-AuNPs. It can be seen from the TEM images that both AuNPs and CYS-AuNPs are spherical, AuNPs are evenly dispersed, and some AuNPs modified with CYS exhibit slight aggregation. The particle size distributions of AuNPs and CYS-AuNPs were analyzed using a dynamic light scattering analyzer (ZS90-DLS, Malvern, UK). According to DLS analysis, as shown in Figure 3b,d, the particle size distribution of AuNPs was concentrated at about 22.48 nm, with an average particle size of 25.21 nm, while the particle size distribution of CYS-AuNPs was concentrated at about 27.55 nm, with an increase in average particle size to 31.59 nm, indicating the effective modification of CYS on AuNPs.

### 3.2. Optimization of NiFe_2_O_4_ Nanofluid Concentration and the Number of Impregnation Times

**Figure 4:**
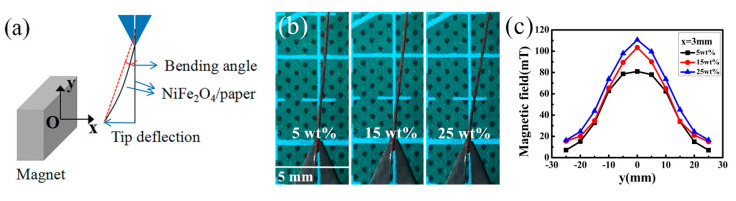
(**a**) Schematic diagram of the testing device; (**b**) Photographs of the response of NiFe_2_O_4_/paper nanocomposites with increased NiFe_2_O_4_ nanofluid concentration; (**c**) Distribution of the magnetic field.

The amount of NiFe_2_O_4_ nanoparticles embedded into the paper was associated with the concentration of NiFe_2_O_4_ nanofluid and the number of times the paper was impregnated by the NiFe_2_O_4_ nanofluid. To improve the sensitivity of the NiFe_2_O_4_/paper nanocomposite, we optimized the working concentration of the NiFe_2_O_4_ nanofluid. Figure 4a is the schematic diagram of the testing device. The tip deflection was measured for the NiFe_2_O_4_/paper impregnated with different concentration of NiFe_2_O_4_ nanofluids as shown in Figure 4b. The NiFe_2_O_4_/paper impregnated with 25 wt% NiFe_2_O_4_ nanofluid had a deflection angle that was significantly larger than that of the paper soaked in 5 wt% and 15 wt% solutions. The mass percentage of NiFe_2_O_4_ nanoparticles was measured in the NiFe_2_O_4_/paper samples as 38.89%, 47.62%, and 52.17%, respectively, for corresponding NiFe_2_O_4_ nanofluid concentrations of 5%, 15%, and 25%. With the NiFe_2_O_4_ nanofluid concentration increased from 5 wt% to 25 wt%, the H around the NiFe_2_O_4_/paper tip continued to increase, as shown in Figure 4c. The result shows that the response reached the largest value of approximately 110.7 mT at a NiFe_2_O_4_ nanofluid concentration of 25 wt%. Since it is difficult to prepare NiFe_2_O_4_ nanofluid at higher mass percentages, 25 wt% was selected as the optimal NiFe_2_O_4_ nanofluid concentration.

**Figure 5:**
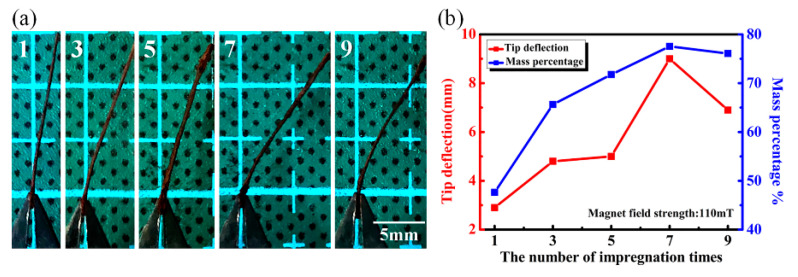
(**a**) Photographs of the response of NiFe_2_O_4_/paper nanocomposites subjected to different rounds of impregnation; (**b**) Tip deflection and mass percentage with increased impregnation.

Next, we optimized the number of times of impregnation with the NiFe_2_O_4_ nanofluid. The NiFe_2_O_4_ nanofluid was used to impregnate the paper 1,3,5,7,9 times, each time for 1 h. The paper was removed from the NiFe_2_O_4_ nanofluid and then dried at 70 °C for 10 min before the next dip. Figure 5a shows the response of NiFe_2_O_4_/paper samples treated with varying times of impregnation. It can be clearly seen that the NiFe_2_O_4_/paper impregnated seven times exhibited the most obvious deflection for H of 110 mT. When the number of impregnations increased from 1 to 9, the tip deflection of NiFe_2_O_4_/paper and the mass percentage of NiFe_2_O_4_ nanoparticles increased first and then decreased, as shown in Figure 5b. The results show the largest response of approximately 9 mm and 77.55% for seven cycles of impregnation. The adsorption capacity of the paper reached saturation and nanoparticles would be lost with increased of impregnation cycles, explaining the decrease in the deflection and mass percentage after nine impregnation steps. Therefore, the optimal impregnation was set as seven times.

### 3.3. Characterization of Paper and NiFe_2_O_4_/Paper Nanocomposite

**Figure 6:**
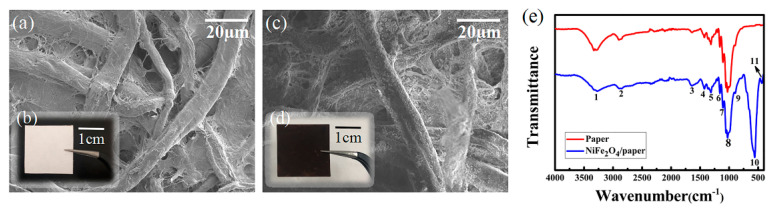
(**a**) SEM image of paper; (**b**) Photograph of paper; (**c**) SEM image of NiFe_2_O_4_/paper nanocomposite; (**d**) Photograph of NiFe_2_O_4_/paper nanocomposite; (**e**) FTIR spectrum of paper and NiFe_2_O_4_/paper nanocomposite. Numbered peaks are described in text and in Table 1.

The paper surface was imaged and appeared smooth (Figure 6b). The surface appearance of the paper and the NiFe_2_O_4_/paper nanocomposite was characterized with a scanning electron microscope (SU3500-SEM, Hitachi, Japan). It can be seen from Figure 6a that the cellulose fibers of the paper appeared intricately overlapped to form a porous and closed cellulose fiber network. The length-diameter ratio of the fibers in the cellulose paper was relatively large, with a diameter of about 10 μm [36]. This indicates that the structure of cellulose paper is stable, providing many attachment sites for NiFe_2_O_4_ nanoparticles. As can be seen from the macrostructure photograph of the NiFe_2_O_4_/paper (Figure 6d), the paper changed from white to brown after impregnating with the NiFe_2_O_4_ nanofluid, and the impregnation is uniform. According to the SEM images (Figure 6c), the NiFe_2_O_4_ nanoparticles were uniformly distributed on the surface of cellulose fibers and diffused into the porous regions of the cellulose fiber networks.

FTIR measurements were conducted using a Fourier-transform infrared spectrometer (Bruker Optik GmbH Tensor 27-FTIR, Ettlingen, Germany). It is evident from the FTIR spectrum presented in Figure 6e that single bond vibration corresponding to cellulose can be detected in analysis of both paper and NiFe_2_O_4_/paper samples. A wide band of 3300 cm^−1^ (1 in Figure 6e) was observed in both samples, which was attributed to the hydrogen bonding of O-H tensile vibration, indicating the hydrophilic tendency of cellulose paper. The band at 2898 cm^−1^ (2) belongs to the aliphatic C-H stretching vibration. The low-intensity bands located at 1642 cm^−1^ (3), 1426 cm^−1^ (4), 1364 cm^−1^ (5), 1158 cm^−1^ (6) and 1107 cm^−1^ (7) are respectively the bending vibration of OH bond in adsorbed water, CH_2_ symmetrical bending, CH bending, C-O-C stretching and reverse bending. The strong band at 1027 cm^−1^ (8) is related to C-C, C-OH and C-H ring and side group vibrations, and is assigned to the amorphous region of cellulose. The small band at 894 cm^−1^ (9) is attributable to C-O-C, C-C-O and C-C-H distortion and stretching vibrations. The band stands for the conformational deformation of the glycoside ^4^C_1_ ring in cellulose and the glycosidic bond between the glucuronic acid ring [37]. In addition, the bands located at 563 cm^−1^ and 416 cm^−1^ (10,11) were assigned to the stretching vibration of the metal oxide bond (Fe-O, Ni-O) at the tetrahedral/octahedral position of the ferrite structure as shown in Figure 6e [38], indicating the successfully immobilization of NiFe_2_O_4_ nanoparticles on the surface of cellulose fibers and diffusion of these nanoparticles into the porous regions among cellulose fiber networks. A summary of infrared band signals associated with bond vibrations within cellulose and metal oxide is highlighted in Table 1. 

### 3.4. Magnet Field Strength Measurement of NiFe_2_O_4_/Paper Nanocomposite

**Figure 7:**
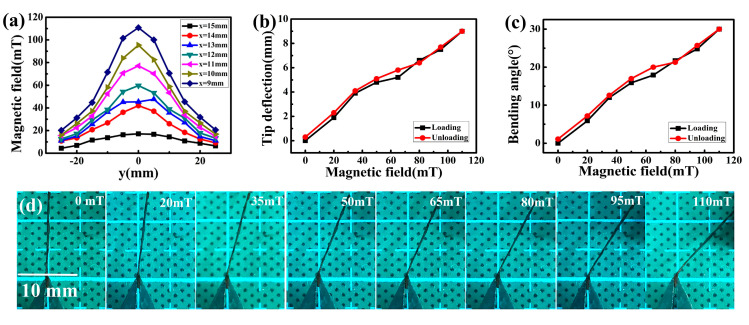
Performance of optimized NiFe_2_O_4_/paper nanocomposite under adjustable magnetic field. (**a**) Distribution of the magnetic field; (**b**) The deflection and (**c**) Bending angle of the sensor tip as functions of magnetic field; (**d**) Images of NiFe_2_O_4_/paper responses with enhanced magnetic field.

Figure 7 presents the performance of the optimized NiFe_2_O_4_/paper nanocomposite with in-plane dimensions of 16 mm × 1 mm under tunable magnetic field. The distribution of the magnetic field was measured by Gauss meter, as presented in Figure 7a. The magnetic intensity decreased with increased distance between the NiFe_2_O_4_/paper and the magnet. The optimized NiFe_2_O_4_/paper were driven by an increasing magnetic intensity, and the snapshots were exhibited in Figure 7d. With the increase of the magnetic field, the deflection and bending angle of the sensor tip increased, reaching peak values at 9 mm and 30° when the magnetic field was 110 mT, as shown in Figure 7b,c. As the magnetic field gradually decreased to 0 mT, the deflection and bending angle of the sensor tip returned to 0.3 mm and 1.1°, which was close to the initial value. The small hysteresis effect may be ascribed to the big flexing deformation in excess of the elastic deformation of NiFe_2_O_4_/paper, resulting in irreversible deformation. 

### 3.5. Hysteresis Loop Measurement of the Biosensor Fabrication Process

**Figure 8:**
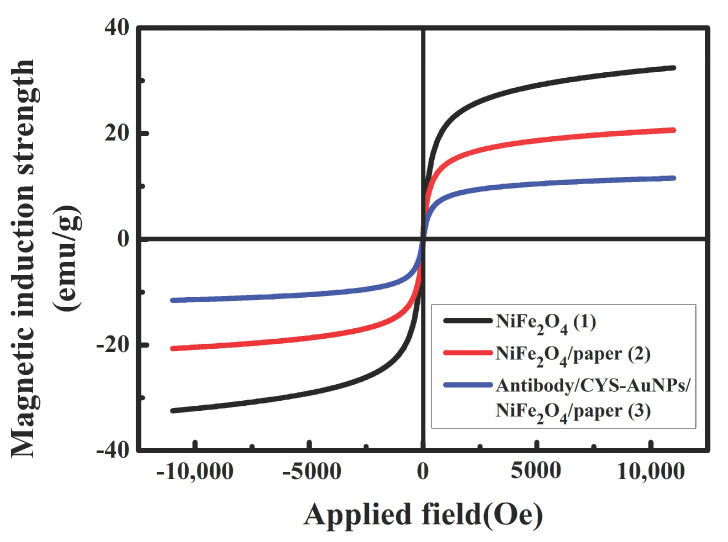
The hysteresis loop of NiFe_2_O_4_ nanoparticles, NiFe_2_O_4_/paper nanocomposite and paper-based ME biosensor (antibody/CYS-AuNPs/NiFe_2_O_4_/paper).

Figure 8 shows the hysteresis loop of NiFe_2_O_4_ nanoparticles, NiFe_2_O_4_/paper nanocomposite, and paper-based ME biosensor. Under an external magnetic field, the B increased with the magnetic field until reaching saturation. The static magnetic permeability values corresponding to each material at the H_m_ were calculated as *μ*_1_ = 29.5 H/m, *μ*_2_ = 18.8 H/m, and *μ*_3_ = 10.5 H/m. Among them, the *B_s_* value of NiFe_2_O_4_ nanoparticles is the largest. Due to the mixing of non-magnetic paper, the *B_s_* of NiFe_2_O_4_/paper decreased, and the corresponding *μ* value also decreased. After immobilization of the antibody on NiFe_2_O_4_/paper, the *μ* value continued to decrease (*μ*_3_ < *μ*_2_ < *μ*_1_) because the antibody tried to expand on the cellulose fiber and cellulose fiber network, but since it was confined, a compressive stress was generated on the surface. At this time, the magnetization direction turned to the direction perpendicular to the stress, and the magnetization in the direction of the compressive stress was weakened, resulting in a weakened *μ* value in the direction of the compressive stress. Analysis of the hysteresis loops confirms the successful fabrication of NiFe_2_O_4_/paper and antibody immobilization.

### 3.6. HSA Detection

**Figure 9:**
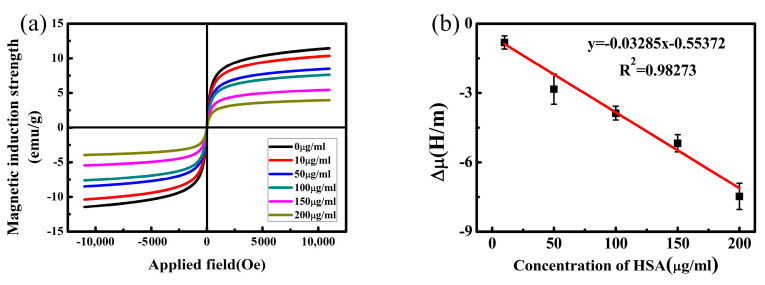
(**a**) The hysteresis loop for HSA detection at different concentrations ranging from 0 μg∙mL^−1^ to 200 μg∙mL^−1^; (**b**) Linear correlation of the Δ*μ* of the biosensor and HSA concentration.

Figure 9a shows the hysteresis loop of the biosensor for HSA detection at concentrations ranging from 0 to 200 μg∙mL^−1^. The specific binding of the antibody and the biomacromolecule HSA caused an increase in the compressive stress on the biosensor, leading to a decrease in the *B_s_* value. The results show that as the HSA concentration increased from 10 μg∙mL^−1^ to 200 μg∙mL^−1^, the *B_s_* value of the biosensor gradually decreased, and the corresponding *μ* at the H_m_ also decreased from 9.4 H/m to 3.5 H/m. By subtracting the μ of the biosensor detecting different concentrations of HSA from the *μ*_3_ (Figure 8) of the biosensor without HSA detection, the corresponding change in static magnetic permeability (Δ*μ*) was obtained, as shown in Figure 9b. The biosensor was measured in 0 μg∙ml^−1^ HSA as a blank control experiment to confirm that the response was specific to HSA. By calculation, the corresponding *μ* at the H_m_ was 10.39 H/m and the Δ*μ* was only 0.11 H/m. This noise response was far less than the Δ*μ* corresponding to 10 μg∙mL^−1^ HSA, so it could be ignored in the detection process. Figure 9b shows that the Δ*μ* of the biosensor shows good linear correlation with HSA concentration for a range of 10 μg∙mL^−1^ to 200 μg∙mL^−1^. The linear equation was y = −0.03285x − 0.55372, and the correlation coefficient was 0.98273, with a detection limit is 0.43 μg∙mL^−1^. The experimental results show that the NiFe_2_O_4_/paper-based ME biosensor can achieve low-cost, accurate and sensitive detection for HSA. The successful detection of HSA demonstrated the unique properties of NiFe_2_O_4_/paper make it an effective material for magnetoelastic biosensor design.

### 3.7. Characterization of the Preparing and Detecting Process

**Figure 10:**
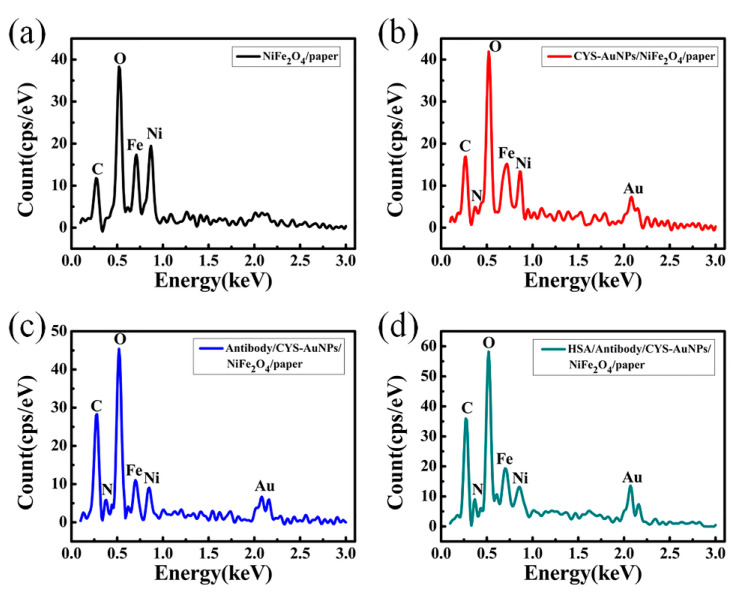
EDS analysis of (**a**) NiFe_2_O_4_/paper nanocomposite; (**b**) paper-based ME sensor (CYS-AuNPs/NiFe_2_O_4_/paper); (**c**) paper-based ME biosensor (Antibody/CYS-AuNPs/NiFe_2_O_4_/paper); (**d**) After HSA detection (HSA/Antibody/CYS-AuNPs/NiFe_2_O_4_/paper).

The surface elements were assessed by energy dispersive spectrometer (SU3500-EDS, Hitachi, Japan). The EDS analysis of non-functionalized NiFe_2_O_4_/paper nanocomposite is shown in Figure 10a. Only C and O elements in cellulose paper and Fe and Ni elements in NiFe_2_O_4_ nanoparticles were detected. During the entire experiment, the amount of Fe and Ni elements showed little reduction, which demonstrated that NiFe_2_O_4_ nanoparticles were firmly attached to the paper and hardly diminished, as shown in Figure 10. The peak corresponding to Au element was detected after deposition of CYS-AuNPs, and the peak of N element in CYS was detected in the range of 0.250–0.5 keV, as shown in Figure 10b, indicating that CYS-AuNPs were successfully deposited on the NiFe_2_O_4_/paper. Since antibodies and HSA are protein molecules, they both contain the C, O, and N elements. After immobilizing the antibody, the peak value of the C, O, and N elements were greater than the peak value of the C, O, and N elements of the paper-based ME sensor, as shown in Figure 10c, indicating effective anti-HSA antibody immobilization. When the antibody-immobilized sensor was exposed to HSA solution (100 μg∙mL^−1^), the peak values of the C, O, and N elements were greatly increased, since the HSA was successfully adsorbed by the specific recognition region of the anti-HSA antibody, as shown in Figure 10d. Comparison of EDS analysis results for the different samples indicates that the fabrication and the detection procedures were successful.

### 3.8. Specificity Measurement

**Figure 11:**
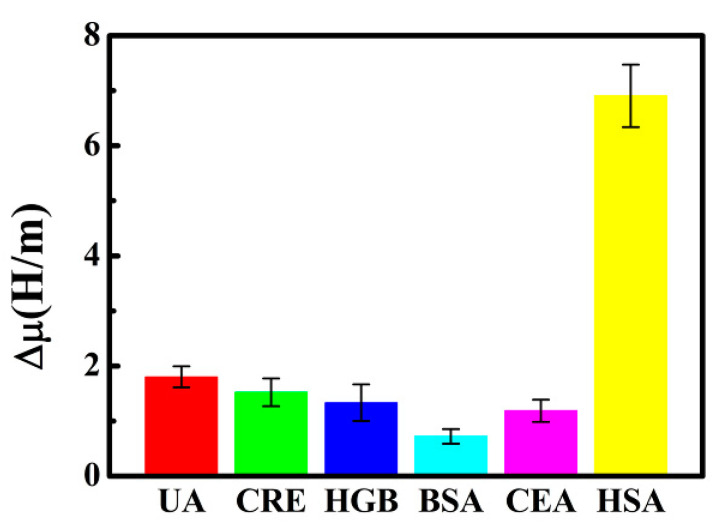
Specificity measurement of the biosensor.

To evaluate the specificity of the paper-based ME biosensor, different potential interferents such as UA, CRE, HGB, BSA, and CEA, with concentrations of 200 μg∙mL^−1^, were detected. It can be clearly observed from Figure 11 that the amount of Δ*μ* caused by HSA interaction is almost four times greater than for other biomolecules, indicating that the biosensor shows little response to these other biomolecules. The results implied that the biosensor has high specificity for the determination of HSA.

## 4. Conclusions

In this study, a NiFe_2_O_4_ nanoparticle-impregnated paper-based ME biosensor for HSA detection was developed based on the magnetoelastic effect of NiFe_2_O_4_ magnetic nanoparticles and the specific binding of antibodies to antigens. The NiFe_2_O_4_/paper was fabricated using a low-cost blending method, and EDS, SEM, and FTIR measurement were used to confirm that the NiFe_2_O_4_ nanoparticles infiltration was successful. The specific binding of HSA and anti-HSA antibody increases the compressive stress on the biosensor surface, resulting in a decrease in the static magnetic permeability. The biosensor can detect a wide range of HSA concentrations (10 μg∙mL^−1^−200 μg∙mL^−1^) and exhibits a linear relationship with a correlation coefficient of 0.98273 within this range. The biosensor detection limit is 0.43 μg∙mL^−1^, meeting the health diagnostic criteria. The NiFe_2_O_4_/paper-based ME biosensor shows a low-cost, specific, and sensitive response towards HSA, making this an effective method for early prevention of diabetes and diabetic complications. Furthermore, this work demonstrated a new and simple method for ME biosensor design by exploiting the unique properties of NiFe_2_O_4_/paper, and provided a new idea for the application of cellulose paper in the field of ME biosensors.

## Figures and Tables

**Table 1:** Infrared band assignments.

Wavenumber Range/cm^−1^	Bonds	Labels
3500–3200	-OH stretching	1
3000–2835	C-H stretching	2
1652–1623	-OH bending of absorbed water	3
1470–1420	C-H bending	4
1391–1361	C-H and C-O vibrations in pyranose ring	5
1260–1150	C-O-C stretching	6
1111–1056	Out-of-phase bending	7
1046–994	C-C, C-OH, C-H ring and side group vibrations	8
898–890	COC, CCO and CCH deformation and stretching	9
565–410	Fe-O, Ni-O stretching	10,11
